# Dietary PUFA Intervention Affects Fatty Acid- and Micronutrient Profiles of Beef and Related Beef Products

**DOI:** 10.3390/foods2030295

**Published:** 2013-07-09

**Authors:** Dirk Dannenberger, Karin Nuernberg, Andrea Herdmann, Gerd Nuernberg, Elke Hagemann, Walter Kienast

**Affiliations:** 1 Institute of Muscle Biology and Growth and Institute of Genetics and Biometry, Leibniz Institute for Farm Animal Biology, 18196 Dummerstorf, Wilhelm-Stahl-Allee 2, Germany; E-Mail: knuernbg@fbn-dummerstorf.de (K.N.); andrea.herdmann@gmx.de (A.H.); gnuernbg@fbn-dummerstorf.de (G.N.); 2 State Office for Agriculture, Food Safety and Fishery, Mecklenburg-Western Pomerania, Department of Contaminant and Residue Analysis, 18059 Rostock, Germany; E-Mail: elke.Hagemann@lallf.mvnet.de; 3 Greifen-Fleisch GmbH, Wolgaster Straße 114, 17489 Greifswald, Germany; E-Mail: kienast@greifen-fleisch.de

**Keywords:** beef, PUFA, beef products, CLA, fat-soluble vitamins, trace elements

## Abstract

The study investigated the dietary impact of 18:3*n*-3 *vs.* 18:2*n*-6 on fatty acid- and micronutrient concentration of beef muscle and the extent of diet- and processing-induced changes of lipid- and micronutrient concentrations of beef products made thereof (German Corned beef (GCB), tea sausage spread (TSS), scalded sausage (SS)). Beef and beef products were obtained from German Holstein bulls which either received a control diet consisting of maize silage and concentrate with soybean meal (41%), or an experimental diet of grass silage and concentrate plus rapeseed cake (12%) and linseed oil (3%). The study revealed that upon an 18:3*n*-3 *vs.* 18:2*n*-6 intervention the amounts of 18:3*n*-3, EPA and Σ*n*-3 LC-PUFA were significantly increased by 2.6, 2.3 and 1.7 fold, respectively. Experimental diet significantly increased β-carotene contents, and the γ-tocopherol contents were decreased. During beef processing, *n*-3 PUFA from beef were found to be product-specifically transferred into the corresponding beef products. 18:3*n*-3 and Σ*n*-3 LC-PUFA contents were found to be 1.4 and 1.5 times higher in GCB from grass silage- than maize silage-fed bulls. The trace element contents in GCB (iron, copper, zinc, selenium) were not affected by the diet; however γ-tocopherol contents were decreased by experimental diet. In conclusion, dietary *n*-3 PUFA were completely transferred into beef products unaffected by beef processing conditions.

## 1. Introduction

In Germany, a slight tendency to higher beef consumption has been seen in recent years (2007–2011) ranging between 8.4 and 9.0 kg (without industrial utilization, losses, bones and diet) per capita [1]. However, the risks associated with the consumption of red meat to human health (e.g., cancer, diabetes and coronary heart diseases) are currently a controversial topic [2,3]. To reduce the risk of cancer, the World Cancer Research Fund report recommends limiting the consumption of red meat to less than 500 g per week [4]. The majority of evidence for the association of red meat with cancer shows an increase in cancer risk for consumers with the highest level of red meat consumption; however, the results of most studies have not reached statistical significance [2]. Additionally, there are disagreements in results’ interpretation with regard to red meat consumption, intake of saturated fatty acids (SFA) and polyunsaturated fatty acids (PUFA) and the recommendations of advisory nutrition committees [5,6]. The German Nutrition Society (DGE) recommends the restriction of red meat consumption, including processed meat, to 600 g per week and a daily fat intake of up to 30% of the total daily energy intake. Less than 10% of this fat intake should be saturated fatty acids (SFAs), approximately 7%–10% should be polyunsaturated fatty acids (PUFAs) and the remaining 10% should be monounsaturated fatty acids (MUFAs) [7]. Red meat is a source of high biological value protein, a significant source of important trace elements, e.g., iron, zinc and selenium, water and fat soluble vitamins (A, B_6_, B_12_, D, and E), and phosphatidylcholine (PC). In red meat, the essential amino acids are well balanced in the ratio required by humans. Lean red meat has a low fat content and therefore is a low-calorie food that contains SFAs (<50% of total fatty acids) and essential *n*-3 and *n*-6 PUFAs that are necessary for human nutrition [2]. The fatty acid composition of adipose and muscle tissues can be affected by factors such as diet, species, fattiness, age/weight, depot site, gender, breed, and season and hormone levels. The fatty acid distribution differs between various tissues, including between intra- and inter-muscular tissue and between abdominal and subcutaneous fat [8]. Levels of long-chain *n*-3 PUFAs in red meat were reported to be effectively elevated upon dietary supplementation with linseed/linseed oil, rapeseed cake/oil or algae or by pasture and grass silage feeding compared to maize silage feeding systems [8,9]. However, the transfer of beneficial long-chain *n*-3 PUFAs into products made from the fresh beef has only sparsely been investigated. To our best knowledge, little information is available about processing-induced changes of fatty acid- and micronutrient concentrations of beef sausages made from fresh meat from cattle fed *n*-3 or *n*-6 PUFA based diets [10,11]. The objective of the present study was to examine the effects of processing-induced changes to beneficial long-chain *n*-3 PUFA, fat soluble vitamins and trace metals concentrations in three different beef products (cooked-, raw- and smoked sausages) made thereof.

## 2. Experimental Section

### 2.1. Experimental Design

A total of 29 German Holstein bulls were randomly selected and assigned one of the two diets: a control diet (*n* = 15) containing maize silage supplemented with concentrate enriched with *n*-6 fatty acids or an experimental diet (*n* = 14) containing grass silage supplemented with concentrate enriched with *n*-3 fatty acids as recently described in detail [9]. The control diet consisted of maize silage and a concentrate (2.5 kg) based on soybean meal (41%), wheat (40%), maize (10%) straw and minerals. The experimental diet consisted of grass silage, concentrate (2.5 kg) based on triticale (40%), wheat (28%), rapeseed cake (13%) and rapeseed oil (2%). The bulls were fed indoor (group keeping) for approximately 240 days and slaughtered at a live weight of 630 kg in the abattoir of the Leibniz Institute for Farm Animal Biology in Dummerstorf (Germany). *Longissimus* muscles were taken 20 min after slaughter for the determination of fatty acids as well as for vitamin and trace element content and stored at −70 °C. All samples were taken from the 6th–13th rib of the right carcass side. 

### 2.2. Sausage Production

Sausages (German Corned beef (GCB), tea sausage spread (TSS), scalded sausage (SS)) were produced by Greifen-Fleisch GmbH (Greifswald, Germany) by the use of lean meat from joint, bug, brisket, hindquarter flank and neck of the slaughtered bulls. Corned beef sausages contained 58% beef (lean meat from joint and bug), 5% beef rind, and drinking water, gelatin, pickling salt, spices, yeast extracts, celeriac, and corn, soy, and plant proteins. The lean meat was scalded until an internal temperature of 68 °C. Then the cooked meat was cooled, minced, and mixed with spices and ingredients. The mass was filled in cleaned guts and scalded. After a central temperature of 78 °C was reached, the sausage was left for another 30 min in the scalding chamber at 82 °C. Tea sausage spread contained 30% beef, hindquarter flank, 20% neck, pork, and pickling salt, spices, sugar and antioxidants. TSS contained in total 94% of beef and small proportion of pork. The meat was fine grinded (2 mm) filled in sausage casing and cold smoked (35 °C). Scalded sausages contained 28% beef, hindquarter and neck, and pork, and pickling salt, spices, sugar and antioxidants. The SS contained in total 83% beef and pork. The raw mixture was fine grinded (3 mm) and filled in sausage casing and hot smoked and scalded (78 °C). Then the sausages were cooled down to 7 °C and storage. Finally, from each carcass of the bulls single sausages were produced, and in total 29 GCB sausages, 29 TSS sausages and 29 SS sausages were analyzed. All sausages were stored at −20 °C until analysis.

### 2.3. Fatty Acid Analysis

Samples of *Longissimus* muscle and sausages were thawed at 4 °C. After homogenisation of approximately 2 g muscle/sausage (Ultra Turrax, IKA, Staufen, Germany; T25, 3 × 15 s, 12,000 rpm) and the addition of the fatty acid C19:0 as an internal standard, the total lipids were extracted in duplicate using chloroform/methanol (2:1, v/v) at room temperature. The detailed sample preparation procedure has been recently described [12]. Briefly, the lipid extracts were esterified by the use of 0.5 M sodium methoxide in methanol and 14% boron trifluoride (BF_3_) in methanol. The fatty acid methyl esters (FAMEs) were stored at −18 °C until used for gas chromatography (GC) analysis. The fatty acid analysis of the muscle lipids was performed using capillary GC with a CP-Sil 88 CB column (100 m × 0.25 mm, Chrompack-Varian, Lake Forest, CA, USA) that was installed in a PerkinElmer gas chromatograph Autosys XL with a flame ionisation detector and split injection (PerkinElmer Instruments, Shelton, CT, USA). The detailed GC conditions were recently described [12]. Briefly, the initial oven temperature was 150 °C, which was held for 5 min; subsequently, the temperature was increased to 175 °C and then to 200 °C at a rate of 2 °C min^−1^ and held for 10 min. Finally, the temperature was increased to 225 °C at a rate of 1.5 °C min^−1^ and held for 25 min. Hydrogen was used as the carrier gas at a flow rate of 1 mL min^−1^. The split ratio was 1:20, and the injector and detector were set at 260 °C and 280 °C, respectively.

### 2.4. Analysis of Conjugated Linoleic Acid (CLA) Isomers

Identification and quantification analysis of the CLA isomers in muscle and sausages fat extracts of the bulls was performed by Ag^+^-ion HPLC involved an HPLC system (LC 10A, Shimadzu, Kyoto, Japan) equipped with a pump (LC-10AD VP), auto sampler (SIL-10AF), 50 µL injection loop, a photodiode array detector (SPD-M 10Avp, Shimadzu, Kyoto, Japan), and a Shimadzu CLASS-VP software system (Version 6.12 SP4). Four ChromSpher 5 Lipids analytical silver ion-impregnated columns (4.6 mm i.d. × 250 mm stainless steel; 5 μm particle size; Agilent, Santa Clara, CA, USA) were used in series. The mobile phase (0.1% acetonitrile in *n*-hexane) was prepared fresh daily and pumped at a flow rate of 1.0 mL/min as described in detail before [13]. The injection volume varied between 20 and 50 µL, according to the content of minor CLA isomers in the different samples. The detector was operated at 233 nm to identify CLA isomers. The identification of CLA isomers was made by the retention time of individual CLA methyl esters (*cis*-9,*trans*-11 CLA, *trans*-9,*trans*-11 CLA, *trans*-10,*cis*-12 CLA, *cis*-9,*trans*-11 CLA, *cis*-9,*cis*-11 CLA and *cis*-11,*trans*-13 CLA). The external calibration plots of the standard solutions were adapted to different concentration levels of individual CLA isomers in the lipid extracts, recently in detail described [13].

### 2.5. Analysis of Fat-Soluble Vitamins

Retinol (vitamin A), β-carotene and tocopherol isomers were extracted according to the methodology recently described by Mahecha *et al.* [14]. Briefly, three subsamples were prepared by homogenising the tissue (4 g per subsample) in 6 mL of a solution of 0.15 M KCl and 0.05 M Tris buffer (pH 7.4) using an Ultra-Turrax disperser (3 × 15 s, 34,000 revolutions per minute, room temperature). The tubes were placed in a water bath (70 °C) and 5 mL of a KOH (potassium hydroxide) solution (10 N) were added. All samples were analysed using an HPLC system (Shimadzu LC-10AD, Kyoto, Japan) equipped with a Shimadzu SIL-10A automatic injector, a Shimadzu SPD-10AV UV-VIS spectrophotometric detector (for retinol (325 nm), and β-carotene (454 nm)) and a Shimadzu RF-10A spectrofluorimetric detector (for α-, γ-, and δ-tocopherol; excitation: 295 nm, emission: 330 nm). Both detectors were used in series. The HPLC column used was a Synergy ODS, 250 × 40 mm (Phenomenex, Torrance, CA, USA). The identification and quantification of the different vitamins was made by the use of an external standard procedure; the external calibration plots were made for a standard mixture in range of 5 to 120 µg/mL. The mobile phase was a mixture of acetonitrile and methanol (3:1 v/v) with a flow rate of 1.5 mL/min and a sample loop of 100 μL.

### 2.6. Analysis of Trace Elements

The determination of selected trace elements, including selenium, copper, iron, and zinc (Se, Cu, Fe, and Zn, respectively), in muscle and GCB samples was performed using an inductively coupled plasma mass spectrometer (ICP-MS 7500ce, Agilent, Santa Clara, CA, USA) as recently described [15]. Briefly, after thawing, the samples were mixed, and approximately 1 g of tissue was treated with 2 mL of HNO_3_ (nitric acid) (65%), 0.5 mL of HCl (37%) and 2 mL of deionised water. The sample preparation was performed using microwave-aided pressure disintegration (CEM, Kamp-Lintfort, Germany). The determination of the selected trace elements was conducted with inductively coupled plasma (ICP-MS 7500ce, Agilent, Santa Clara, CA, USA). Trace elements were analysed twice for each sample and expressed as mg/kg of fresh muscle/sausage.

### 2.7. Reagents and Chemicals

The reference standard F.A.M.E. Mix C4-C24 was obtained from Sigma-Aldrich (Deisenhofen, Germany) for use in the fatty acid analyses. Additionally, individual methyl esters of 18:4*n*-3, 22:4*n*-6 and 22:5*n*-3 was purchased from Matreya (Pleasant Gap, PA, USA). Methyl esters of 18:1*trans*-11 and 18:1*cis*-11 was purchased from Larodan Fine Chemicals (Malmö, Sweden). All individual reference standards for the analysis of fat-soluble vitamins and trace elements were purchased from Sigma-Aldrich (Deisenhofen, Germany). All solvents and other chemicals used for GC and HPLC (high-performance liquid chromatography) were of HPLC grade and obtained from Lab-Scan (Dublin, Ireland).

### 2.8. Statistical Analysis

The effects of diet were estimated using the GLM procedure of the SAS software system (SAS © Systems, Release 9.2, SAS Institute Inc., Cary, NC, USA). All tables indicate the least squares mean (LSM) and the standard error (SEM) of the LSMs. All post hoc tests were performed at a significance level of *p* ≤ 0.05 using the Tukey-Kramer correction for multiple tests.

## 3. Results and Discussions

The concentrations (mg/100g muscle/sausage) of selected fatty acids, total SFA, total monounsaturated fatty acids (MUFA) and total PUFA in fresh beef and products made thereof (German Corned beef (GCB), tea sausage spread (TSS), scalded sausage (SS)) are presented in Table 1, Table 2, Table 3, Table 4. Diet caused significant changes in fresh muscle fatty acid composition. Beef muscle from bulls fed with grass silage-based diet was clearly superior with regard to higher concentration of beneficial PUFA and lower SFA concentrations in comparison to bulls fed maize silage-based diet. The concentrations of 18:3*n*-3, eicosapentaenoic acid (EPA, 20:5*n*-3), docosapentaenoic acid (DPA, 22:5*n*-3), docosahexaenoic acid (DHA, 22:6*n*-3), and total *n*-3 PUFA were significantly increased in fresh beef of grass silage-fed bulls (Table 1). These higher amounts of *n*-3 PUFA caused a reduced *n*-6/*n*-3 PUFA ratio (2.3:1) compared to the ratio in fresh beef of maize silage-fed bulls (5.8:1). This low *n*-6/*n*-3 PUFA ratio corresponds to the recommendation of the German Nutrition Society (*n*-6/*n*-3 PUFA ratio ≤ 5:1) [7]. The European Food and Safety Authority (EFSA) determined that 250 mg should be the labelling reference intake value for LC *n*-3 fatty acids—most notably eicosapentaenoic acid and docosahexaenoic acid [16]. EFA suggested that foods containing 15% (of 250 mg EPA + DHA) are labelled “Source of” and 30% (of 250 mg EPA+DHA) are labelled “High in” [17]. A 250 g serving of muscle of grass silage-fed bulls contains 38 mg EPA + DHA and consequently a source of LC *n*-3 PUFA. Additionally, the concentrations of SFA, 12:0, 14:0 and 16:0 were significantly decreased in fresh beef by grass silage-based diet compared to maize silage-based diet (Table 1). Higher consumption of mainly of 12:0, 14:0 and 16:0 increases levels of low-density lipoprotein (LDL) cholesterol and has been positively associated with risk of cardiovascular diseases (CVD) [5]. In the literature, investigations of the transfer from beneficial fatty acids in fresh meat from beef cattle fed *n*-3 and/or *n*-6 PUFA-based diets into beef sausages produced thereof, including changes during processing, were only sparsely described [9]. The fat content of Corned beef (GCB), a cooked sausage, was low (2.0%–2.2 %, Table 2) and similar compared to fresh meat (2.1%–2.8%). Therefore it can be considered as a low-fat beef product according to the DGE recommendations [7]. The concentrations of single and total *n*-3 fatty acids in GCB of bulls fed grass silage-based diet were found to be 1.4 and 1.5 times higher compared to GCB of maize silage-fed bulls, resulting in a complete transfer from muscle to beef product unaffected by processing conditions (Table 2). The concentrations of *n*-6 PUFA and SFA in GCB were not significantly affected by the diet.

Based on this, the *n*-6/*n*-3 PUFA ratio in GCB of (4.0:1) from bulls fed grass silage-based diet was lower compared to the ratio in GCB of maize silage fed bulls and meets the recommendation of the German Nutrition Society (DGE). Higher single and total *n*-3 PUFA concentrations were detected in TSS and SS mainly based on much higher fat contents, 21.1%–21.8 % and 17.8%–18.5 % respectively, compared to GCB (Table 3, Table 4). Also, in TSS the single and total *n*-3 PUFA concentrations were significantly increased in sausages of grass silage-based fed bulls compared to maize silage fed bulls. The total *n*-3 PUFA were detected up to 289 mg/100 g TSS (Table 3). Comparable to GCB, TSS from grass silage-based fed bulls was shown to completely transfer *n*-3 PUFA from muscle to beef product unaffected by processing conditions. Total SFA and PUFA concentrations in TSS were not affected by the diet; however the *n*-6/*n*-3 PUFA ratio was significantly decreased in TSS of grass silage-fed bulls compared to this ratio in TSS of maize silage-fed bulls based on higher *n*-3 PUFA concentration and unchanged *n*-6 PUFA level in TSS of grass silage-fed bulls (Table 3). In SS as well as single and some fatty acids were not diet affected (Table 4). The reason was the higher proportion of pork compared to beef necessary to produce this kind of smoked sausage, resulting in overlapping the transfer of beneficial *n*-3 PUFA and other bioactive fatty acids from fresh beef to the sausages in case of SS.

**Table 1:** Fatty acid concentration (mg/100 g) in *Longissimus* muscle of German Holstein bulls fed a different diet [16].

Fatty acids (mg/100 g)	Control (*n* = 15) LSM_SEM_	Experiment (*n* = 14) LSM_SEM_	Significance
Sum fatty acids	2367_182_^a^	1764_188_^b^	0.029
C12:0	1.5_0.13_^a^	1.0_0.14_^b^	0.031
C14:0	63.4_6.04_^a^	42.6_6.25_^b^	0.017
C16:0	627.2_52.0_^a^	448.1_53.8_^b^	0.020
C16:1	89.2_8.05_^a^	57.9_8.33_^b^	0.013
C18:0	342.6_25.6_^a^	277.3_26.5_^b^	0.086
C18:1*trans-*11	13.6_1.18_	13.9_1.22_	0.862
C18:1*cis-*9	892.8_76.3_^a^	614.9_79.0_^b^	0.018
C18:1*cis-*11	28.8_2.14_	22.7_2.22_	0.057
C18:2*n*-6	112.9_3.33_^a^	95.2_3.44_^b^	<0.001
C18:3*n*-3	12.9_1.08_^a^	33.4_1.11_^b^	<0.001
C20:4*n*-6	29.9_0.95_^a^	26.4_0.99_^b^	0.015
C20:5*n*-3	3.8_0.30_^a^	8.8_0.32_^b^	<0.001
C22:4*n*-6	4.6_0.14_^a^	2.5_0.15_^b^	<0.001
C22:5*n*-3	8.4_0.29_^a^	12.0_0.31_^b^	<0.001
C22:6*n*-3	1.0_0.05_^a^	1.4_0.05_^b^	<0.001
Sum SFA	1078_84.8_^a^	805.5_87.7_^b^	0.029
Sum UFA	1272_95.5_^a^	943.9_98.9_^b^	0.023
Sum MUFA	1083_92.1_^a^	752.0_95.3_^b^	0.019
Sum PUFA	187.8_5.61_	191.9_5.81_	0.613
Sum *n*-3 FA	27.5_1.35_^a^	56.5_1.40_^b^	<0.001
Sum *n*-6 FA	157.6_4.28_^a^	131.5_4.43_^b^	<0.001
Ratio *n*-6/*n*-3 FA	5.8_0.13_^a^	2.3_0.13_^b^	<0.001

Different small letters (a, b) denote significant effect of diet groups (*p* ≤ 0.05); FA, fatty acids; Total SFA: 10:0 + 11:0 + 12:0 + 13:0 + 14:0 + 15:0 + 16:0 + 17:0 + 18:0 + 20:0 + 21:0 + 22:0 + 23:0 + 24:0; Total UFA: 14:1 + 15:1 + 16:1 + 17:1 + 18:1*t* + 18:1*c*9 + C18:1*c*11 + C22:1 + C24:1 +: 18:2*t + * 18:2*n-*6 + 18:3*n-*3 + 18:4*n*-3 + 20:3*n*-6 + 20:4*n-*6 + 20:5*n-*3 + 22:1 + 22:4*n-*6 + 22:5*n-*3 + 22:6*n*-3 + *c*9,*tr*11CLA + 18:3*n*-6 + 20:2*n*-6 + 20:3*n*-3 + 22:2*n*-6; Total MUFA: 14:1 + 15:1 + 16:1 + 17:1 + 18:1*t* + 18:1*c*9 + C18:1*c*11 + C22:1 + C24:1; Total PUFA: 18:2*t +* 18:2*n-*6 + 18:3*n-*3 + 18:4*n*-3 + 20:3*n*-6 + 20:4*n-*6 + 20:5*n-*3 + 22:1 + 22:4*n-*6 + 22:5*n-*3 + 22:6*n*-3 + *c*9,*tr*11CLA + 18:3*n*-6 + 20:2*n*-6 + 20:3*n*-3 + 22:2*n*-6; Total *n*-3 PUFA: 20:3*n*-3 + 22:6*n*-3 + 22:5*n*-3 + 20:5*n*-3 + 18:4*n*-3 + 18:3*n*-3; Total *n*-6 PUFA: 22:2*n*-6 + 20:2*n*-6 + 18:3*n*-6 + 22:4*n*-6 + 20:3*n*-6 + 18:2*n*-6 + 20:4*n*-6.

**Table 2:** Fatty acid concentration (mg/100 g) of corned beef produced from meat sections of German Holstein bulls fed a different diet.

Fatty acids (mg/100 g)	Control (*n* = 15) LSM_SEM_	Experiment (*n* = 14) LSM_SEM_	Significance
Sum fatty acids	2165_118_	1966_122_	0.250
C12:0	2.1_0.55_	2.4_0.57_	0.646
C14:0	39.4_2.47_	33.9_2.55_	0.131
C16:0	491.4_28.1_	435.3_29.1_	0.177
C16:1	81.9_5.17_^a^	67.2_5.36_^b^	0.057
C18:0	297.6_16.2_	290.3_16.8_	0.759
C18:1*trans-*11	15.7_1.43_	15.4_1.48_	0.859
C18:1*cis-*9	840.2_49.6_	738.7_51.4_	0.167
C18:1*cis-*11	38.5_2.96_	34.2_3.07_	0.317
C18:2*n*-6	167.9_14.8_	150.8_15.3_	0.427
C18:3*n*-3	21.3_2.83_^a^	29.4_2.90_^b^	0.059
C20:4*n*-6	28.2_1.73_	28.6_1.79_	0.886
C20:5*n*-3	4.0_0.68_^a^	7.5_0.70_^b^	0.001
C22:4*n*-6	4.8_0.29_^a^	3.5_0.30_^b^	0.003
C22:5*n*-3	8.9_0.73_^a^	12.1_0.75_^b^	0.005
C22:6*n*-3	1.1_0.08_^a^	1.5_0.08_^b^	0.003
Sum SFA	867.5_47.3_	799.3_49.0_	0.325
Sum UFA	1298_72.1_	1167_74.7_	0.217
Sum MUFA	1024_59.9_	898.0_62.0_	0.155
Sum PUFA	253.1_18.0_	248.6_18.7_	0.866
Sum *n*-3 FA	38.2_4.14_^a^	53.3_4.28_^b^	0.017
Sum *n*-6 FA	213.6_15.7_	194.1_16.2_	0.393
Ratio *n*-6/*n*-3 FA	5.8_0.37_^a^	4.0_0.38_^b^	0.002

Different small letters (a, b) denote significant effect of diet groups (*p* ≤ 0.05); FA, fatty acids; Total SFA: 10:0 + 11:0 + 12:0 + 13:0 + 14:0 + 15:0 + 16:0 + 17:0 + 18:0 + 20:0 + 21:0 + 22:0 + 23:0 + 24:0; Total UFA: 14:1 + 15:1 + 16:1 + 17:1 + 18:1t + 18:1*c*9 + C18:1*c*11 + C22:1 + C24:1 +: 18:2*t +* 18:2*n-*6 + 18:3*n-*3 + 18:4*n*-3 + 20:3*n*-6 + 20:4*n-*6 + 20:5*n-*3 + 22:1 + 22:4*n-*6 + 22:5*n-*3 + 22:6*n*-3 + *c*9,*tr*11CLA + 18:3*n*-6 + 20:2*n*-6 + 20:3*n*-3 + 22:2*n*-6; Total MUFA: 14:1 + 15:1 + 16:1 + 17:1 + 18:1t + 18:1*c*9 + C18:1*c*11 + C22:1 + C24:1; Total PUFA: 18:2*t +* 18:2*n-*6 + 18:3*n-*3 + 18:4*n*-3 + 20:3*n*-6 + 20:4*n-*6 + 20:5*n-*3 + 22:1 + 22:4*n-*6 + 22:5*n-*3 + 22:6*n*-3 + *c*9,*tr*11CLA + 18:3*n*-6 + 20:2*n*-6 + 20:3*n*-3 + 22:2*n*-6; Total *n*-3 PUFA: 20:3*n*-3 + 22:6*n*-3 + 22:5*n*-3 + 20:5*n*-3 + 18:4*n*-3 + 18:3*n*-3; Total *n*-6 PUFA: 22:2*n*-6 + 20:2*n*-6 + 18:3*n*-6 + 22:4*n*-6 + 20:3*n*-6 + 18:2*n*-6 + 20:4*n*-6.

**Table 3:** Fatty acid concentration (mg/100 g) of tea sausages spread produced from meat sections of German Holstein bulls fed a different diet.

Fatty acids (mg/100 g)	Control (*n* = 15) LSM_SEM_	Experiment (*n* = 14) LSM_SEM_	Significance
Sum fatty acids	21777_599_	21836_620_	0.945
C12:0	18.8_1.15_	20.0_1.19_	0.473
C14:0	462.8_12.9_	439.9_13.3_	0.227
C16:0	5367_139_	5283_144_	0.676
C16:1	789.5_23.6_^a^	716.7_24.5_^b^	0.041
C18:0	3355_107_	3486_111_	0.402
C18:1*trans-*11	108.3_7.32_	97.4_7.57_	0.312
C18:1*cis-*9	8311_263_	8350_272_	0.918
C18:1*cis-*11	405.2_16.5_	404.4_17.1_	0.976
C18:2*n*-6	1636_94.3_	1683_97.6_	0.731
C18:3*n*-3	176.8_13.5_^a^	230.2_14.0_^b^	0.011
C20:4*n*-6	45.5_2.35_	45.9_2.43_	0.897
C20:5*n*-3	5.3_0.32_^a^	6.2_0.33_^b^	0.059
C22:4*n*-6	17.2_0.89_	16.0_0.92_	0.343
C22:5*n*-3	17.8_0.81_^a^	20.4_0.84_^b^	0.036
C22:6*n*-3	5.3_0.49_	5.4_0.51_	0.893
Sum SFA	9523_241_	9552_250_	0.935
Sum UFA	12253_389_	12284_403_	0.957
Sum MUFA	10173_303_	10092_314_	0.855
Sum PUFA	2032_112_	2145_116_	0.486
Sum *n*-3 FA	228.1_15.3_^a^	289.2_15.8_^b^	0.010
Sum *n*-6 FA	1782_101_	1833_104_	0.730
Ratio *n*-6/*n*-3 FA	7.95_0.33_^a^	6.37_0.34_^b^	0.003

Different small letters (a, b) denote significant effect of diet groups (*p* ≤ 0.05); FA, fatty acids; Total SFA: 10:0 + 11:0 + 12:0 + 13:0 + 14:0 + 15:0 + 16:0 + 17:0 + 18:0 + 20:0 + 21:0 + 22:0 + 23:0 + 24:0; Total UFA: 14:1 + 15:1 + 16:1 + 17:1 + 18:1t + 18:1*c*9 + C18:1*c*11 + C22:1 + C24:1 + : 18:2*t +* 18:2*n-*6 + 18:3*n-*3 + 18:4*n*-3 + 20:3*n*-6 + 20:4*n-*6 + 20:5*n-*3 + 22:1 + 22:4*n-*6 + 22:5*n-*3 + 22:6*n*-3 + *c*9,*tr*11CLA + 18:3*n*-6 + 20:2*n*-6 + 20:3*n*-3 + 22:2*n*-6; Total MUFA: 14:1 + 15:1 + 16:1 + 17:1 + 18:1t + 18:1*c*9 + C18:1*c*11 + C22:1 + C24:1; Total PUFA: 18:2*t +* 18:2*n-*6 + 18:3*n-*3 + 18:4*n*-3 + 20:3*n*-6 + 20:4*n-*6 + 20:5*n-*3 + 22:1 + 22:4*n-*6 + 22:5*n-*3 + 22:6*n*-3 + *c*9,*tr*11CLA + 18:3*n*-6 + 20:2*n*-6 + 20:3*n*-3 + 22:2*n*-6; Total *n*-3 PUFA: 20:3*n*-3 + 22:6*n*-3 + 22:5*n*-3 + 20:5*n*-3 + 18:4*n*-3 + 18:3*n*-3; Total *n*-6 PUFA: 22:2*n*-6 + 20:2*n*-6 + 18:3*n*-6 + 22:4*n*-6 + 20:3*n*-6 + 18:2*n*-6 + 20:4*n*-6.

**Table 4:** Fatty acid concentration (mg/100 g) of scalded sausage produced from meat sections of German Holstein bulls fed a different diet.

Fatty acids (mg/100 g)	Control (*n* = 15) LSM_SEM_	Experiment (*n* = 14) LSM_SEM_	Significance
Sum fatty acids	17845_751_	18585_778_	0.500
C12:0	21.8_2.76_	22.2_2.86_	0.920
C14:0	307.0_13.4_	314.6_13.9_	0.698
C16:0	4415_178_	4606_184_	0.463
C16:1	483.1_22.6_	467.7_23.4_	0.640
C18:0	2479_119_	2648_123_	0.331
C18:1*trans-*11	55.1_7.38_	52.9_7.64_	0.833
C18:1*cis-*9	6641_279_	6878_288_	0.560
C18:1*cis-*11	525.6_23.3_	501.3_24.1_	0.477
C18:2*n*-6	1909_124_	2058_129_	0.411
C18:3*n*-3	155.7_11.2_	180.3_11.6_	0.139
C20:4*n*-6	77.3_2.99_	74.8_3.10_	0.558
C20:5*n*-3	7.3_0.44_	8.2_0.46_	0.151
C22:4*n*-6	25.7_1.69_	22.3_1.74_	0.181
C22:5*n*-3	27.9_2.01_	27.3_2.08_	0.835
C22:6*n*-3	8.1_0.86_	7.4_0.89_	0.589
Sum SFA	7397_313_	7773_324_	0.410
Sum UFA	10449_453_	10812_469_	0.582
Sum MUFA	8061_324_	8255_335_	0.681
Sum PUFA	2364_150_	2533_156_	0.444
Sum *n*-3 FA	228.2_15.7_	253.2_16.3_	0.279
Sum *n*-6 FA	2125_136_	2267_140_	0.472
Ratio *n*-6/*n*-3 FA	9.4_0.22_	9.0_0.23_	0.230

FA, fatty acids; Total SFA: 10:0 + 11:0 + 12:0 + 13:0 + 14:0 + 15:0 + 16:0 + 17:0 + 18:0 + 20:0 + 21:0 + 22:0 + 23:0 + 24:0; Total UFA: 14:1 + 15:1 + 16:1 + 17:1 + 18:1t + 18:1*c*9 + C18:1*c*11 + C22:1 + C24:1 + 18:2*t + * 18:2*n-*6 + 18:3*n-*3 + 18:4*n*-3 + 20:3*n*-6 + 20:4*n-*6 + 20:5*n-*3 + 22:1 + 22:4*n-*6 + 22:5*n-*3 + 22:6*n*-3 + *c*9,*tr*11CLA + 18:3*n*-6 + 20:2*n*-6 + 20:3*n*-3 + 22:2*n*-6; Total MUFA: 14:1 + 15:1 + 16:1 + 17:1 + 18:1t + 18:1*c*9 + C18:1*c*11 + C22:1 + C24:1; Total PUFA: 18:2*t +* 18:2*n-*6 + 18:3*n-*3 + 18:4*n*-3 + 20:3*n*-6 + 20:4*n-*6 + 20:5*n-*3 + 22:1 + 22:4*n-*6 + 22:5*n-*3 + 22:6*n*-3 + *c*9,*tr*11CLA + 18:3*n*-6 + 20:2*n*-6 + 20:3*n*-3 + 22:2*n*-6; Total *n*-3 PUFA: 20:3*n*-3 + 22:6*n*-3 + 22:5*n*-3 + 20:5*n*-3 + 18:4*n*-3 + 18:3*n*-3; Total *n*-6 PUFA: 22:2*n*-6 + 20:2*n*-6 + 18:3*n*-6 + 22:4*n*-6 + 20:3*n*-6 + 18:2*n*-6 + 20:4*n*-6.

Beside PUFA another group of fatty acids, conjugated linoleic acids (CLA), has been identified with biological properties which may potentially alter the risk of developing metabolic disorders including diabetes and cardiovascular diseases (CVD) [18,19]. To date, animal and human studies have indicated that *cis-*9,*trans*-11 CLA and *trans*-10,*cis*-12 CLA, show biological activity including prevention of different types of cancer, cardiovascular health, decreasing body fat, and improved immune response predominantly in animal models; however in recent human studies inconsistent effects were reported [19,20]. There is much evidence that the physiological properties of CLA are isomer specific partly with opposing effects. The concentrations of selected CLA isomers in muscle and sausages are shown in Table 5.

**Table 5:** Conjugated linoleic acid (CLA) isomer concentrations (mg/100 g) in muscle and sausages from German Holstein bulls fed different diets (different letters (a, b) denote significant effect of diet).

CLA isomer (mg/100 g)	Control (*n* = 15) LSM_SEM_	Experiment (*n* = 14) LSM_SEM_	Significance
*Muscle*			
*trans*-12,*trans*-14 CLA	0.06_0.01_^a^	0.18_0.01_^b^	<0.001
*trans*-11,*trans*-13 CLA	0.10_0.02_^a^	0.35_0.02_^b^	<0.001
*trans*-10,*trans*-12 CLA	0.06_0.005_	0.05_0.005_	0.095
*trans*-9,*trans*-11 CLA	0.13_0.01_^a^	0.15_0.01_^b^	0.043
*trans*-11,*cis*-13 CLA	0.16_0.02_^a^	0.58_0.02_^b^	<0.001
*trans*-10,*cis*-12 CLA	0.25_0.02_^a^	0.17_0.02_^b^	0.007
*cis*-9,*trans*-11 CLA	4.05_0.21_	4.22_0.22_	0.580
*trans*-7,*cis*-9 CLA	0.95_0.05_	0.80_0.05_	0.066
*Corned beef*			
*trans*-12,*trans*-14 CLA	0.04_0.01_^a^	0.09_0.01_^b^	<0.001
*trans*-11,*trans*-13 CLA	0.06_0.02_^a^	0.18_0.02_^b^	<0.001
*trans*-10,*trans*-12 CLA	0.04_0.003_	0.05_0.003_	0.381
*trans*-9,*trans*-11 CLA	0.13_0.01_	0.16_0.01_	0.110
*trans*-11,*cis*-13 CLA	0.13_0.04_^a^	0.58_0.05_^b^	<0.001
*trans*-10,*cis*-12 CLA	0.22_0.02_^a^	0.17_0.02_^b^	0.025
*cis*-9,*trans*-11 CLA	3.74_0.30_	4.07_0.31_	0.454
*trans*-7,*cis*-9 CLA	0.77_0.05_	0.78_0.06_	0.933
*Tea sausage spread*			
*trans*-12,*trans*-14 CLA	0.77_0.13_^a^	1.90_0.14_^b^	<0.001
*trans*-11,*trans*-13 CLA	0.96_0.15_^a^	2.78_0.16_^b^	<0.001
*trans*-10,*trans*-12 CLA	0.76_0.09_	0.76_0.10_	0.959
*trans*-9,*trans*-11 CLA	1.63_0.21_	2.12_0.22_	0.119
*trans*-11,*cis*-13 CLA	1.05_0.21_^a^	4.15_0.22_^b^	<0.001
*trans*-10,*cis*-12 CLA	2.04_0.13_^a^	1.09_0.14_^b^	<0.001
*cis*-9,*trans*-11 CLA	33.15_3.31_	40.06_3.43_	0.159
*trans*-7,*cis*-9 CLA	6.23_0.61_	6.39_0.63_	0.862
*Scalded sausages*			
*trans*-12,*trans*-14 CLA	0.38_0.09_^a^	0.82_0.10_^b^	0.004
*trans*-11,*trans*-13 CLA	0.35_0.11_^a^	0.88_0.12_^b^	0.003
*trans*-10,*trans*-12 CLA	0.29_0.04_	0.35_0.04_	0.325
*trans*-9,*trans*-11 CLA	1.31_0.15_	1.38_0.16_	0.768
*trans*-11,*cis*-13 CLA	1.02_0.33_^a^	3.53_0.34_^b^	<0.001
*trans*-10,*cis*-12 CLA	3.40_0.27_^a^	2.25_0.28_^b^	0.006
*cis*-9,*trans*-11 CLA	18.48_1.50_	17.80_1.55_	0.756
*trans*-7,*cis*-9 CLA	5.18_0.32_	4.55_0.33_	0.175

FA, fatty acids; Total SFA: 10:0 + 11:0 + 12:0 + 13:0 + 14:0 + 15:0 + 16:0 + 17:0 + 18:0 + 20:0 + 21:0 + 22:0 + 23:0 + 24:0; Total UFA: 14:1 + 15:1 + 16:1 + 17:1 + 18:1t + 18:1*c*9 + C18:1*c*11 + C22:1 + C24:1 + 18:2*t +* 18:2*n-*6 + 18:3*n-*3 + 18:4*n*-3 + 20:3*n*-6 + 20:4*n-*6 + 20:5*n-*3 + 22:1 + 22:4*n-*6 + 22:5*n-*3 + 22:6*n*-3 + *c*9,*tr*11CLA + 18:3*n*-6 + 20:2*n*-6 + 20:3*n*-3 + 22:2*n*-6; Total MUFA: 14:1 + 15:1 + 16:1 + 17:1 + 18:1t + 18:1*c*9 + C18:1*c*11 + C22:1 + C24:1; Total PUFA: 18:2*t +* 18:2*n-*6 + 18:3*n-*3 + 18:4*n*-3 + 20:3*n*-6 + 20:4*n-*6 + 20:5*n-*3 + 22:1 + 22:4*n-*6 + 22:5*n-*3 + 22:6*n*-3 + *c*9,*tr*11CLA + 18:3*n*-6 + 20:2*n*-6 + 20:3*n*-3 + 22:2*n*-6; Total *n*-3 PUFA: 20:3*n*-3 + 22:6*n*-3 + 22:5*n*-3 + 20:5*n*-3 + 18:4*n*-3 + 18:3*n*-3; Total *n*-6 PUFA: 22:2*n*-6 + 20:2*n*-6 + 18:3*n*-6 + 22:4*n*-6 + 20:3*n*-6 + 18:2*n*-6 + 20:4*n*-6.

The concentrations of selected CLA isomers in muscle and sausages are shown in Table 5. The main CLA isomer, *cis*-9,*trans*-11CLA, was detected in highest concentration in sausages and tended to higher values in sausages of grass silage-fed bulls. Some minor isomers detected in higher concentration in muscle of grass silage-fed bulls (*trans*-11,*trans*-13 CLA, *trans*-9,*trans*-11 CLA, *trans*-11,*cis-*13 CLA) occurred in higher concentrations in GCB, TSS and partly in SS compared to products of maize silage-fed bulls, also. The highest values up to 40.1 mg/100 g sausage were measured in TSS related to the highest fat content of 21.8 % (Table 5). It can be concluded that *n*-3 PUFA, CLA isomers detected in fresh beef can be successfully transferred to meat products unaffected by processing conditions. 

**Figure 1:**
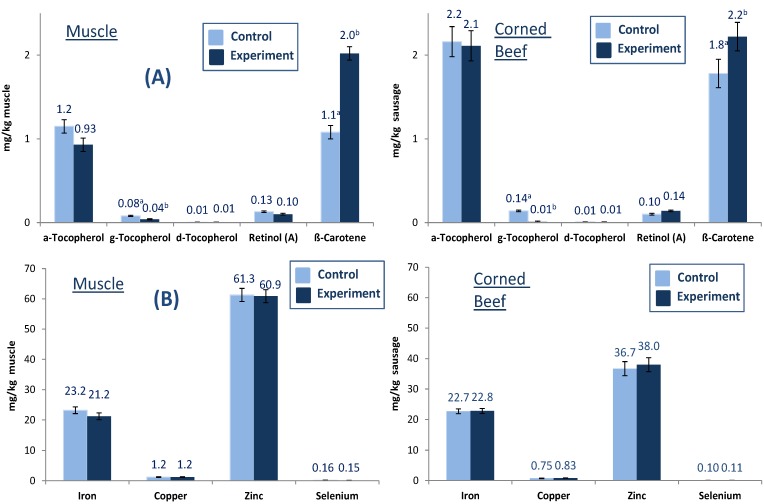
Fat soluble vitamin (**A**) and trace metal (**B**) concentrations (mg/kg) in fresh muscle and beef product (GCB) from German Holstein bulls fed different diets (different letters (a, b) denote significant effect of diet).

In addition to fatty acids, the concentrations of fat-soluble vitamins and trace elements in fresh meat and one selected sausage (GCB) were investigated. Much interest has been focused on the protection of *n*-3 PUFA, also in beef, for human consumption because these PUFA are highly susceptible to lipid peroxidation by highly reactive species (ROS) originating from endogenous and exogenous sources. Antioxidants such as vitamin E protect cells against attacks of ROS. Vitamin E is a fat-soluble vitamin existing in eight different isoforms with various antioxidant activities; the most active one is α-tocopherol [21]. The fat-soluble vitamin concentrations in fresh meat and one selected sausage (GCB) are presented in Figure 1A,B). Diet effected lipid soluble vitamins in fresh beef. Whereas the concentration of β-carotene significantly increased in fresh beef of grass silage-fed bulls, the concentration of the major vitamin E homologue α-tocopherol tended to decrease ranging between 0.9 and 1.2 mg/g muscle, while there was no effect on retinol (Figure 1A). δ-Tocopherol did not change in treatment animals while γ-tocopherol significantly decreased. The antioxidants predominantly occurred in pasture/grass silage caused higher levels of vitamin E and β-carotene in cattle with benefits of lower lipid peroxidation and better color stability despite higher level of highly susceptible *n*-3 PUFA [21].

The concentrations of selected trace elements (iron, copper zinc and selenium) in fresh muscle and GCB are presented in Figure 1B. The iron and zinc concentrations in muscle and sausage ranged between 21.2 and 23.2 mg/kg muscle and 36.7 and 61.3 mg/kg muscle, respectively. Diet did not show significant effects in muscle and GCB for the trace elements iron, copper, zinc and selenium.

## 4. Conclusions

Beneficial fatty acids (*n*-3 PUFA, CLAs) in fresh beef enriched by long term feeding of *n*-3 and/or *n*-6 PUFA can be successfully transferred into different beef products (cold-, raw- and smoked sausages) manufactured under various production conditions. In addition, the present study revealed that such meat accumulated beneficial micronutrients providing a good source of bioactive fatty acids, vitamins and trace elements that are essential for human nutrition.
